# An audit of liaison service provision in Aneurin Bevan University Health Board

**DOI:** 10.1192/bjo.2021.297

**Published:** 2021-06-18

**Authors:** Jennifer Rankin, Heledd Espley

**Affiliations:** Aneurin Bevan University Health Board

## Abstract

**Aims:**

Aneurin Bevan University Health Board (ABUHB) isundertaking a review of the Mental Health Liaison Service provision within it's acute general hospitals. The current liaison service is a small nurse led team which is available between 8am and midnight. ABUHB has recently opened a new Specialist Critical Care Hospital with the liaison service moving into a new base. A new model of care has been developed across the healthboard which has stretched the Liaison Service across several sites. Therefore, the liaison service may need to expanded to be able to provide high quality and timely care across a wide geographical area. The audit aims to idenitfy areas in which the liaison service is performing well in while identiying areas that need improvement. This audit may provide a focus for recommendations to enhance the current liaison provision.

**Method:**

The liaison service was audited against RCPsych Psychiatric Liaison Accreditation Network (PLAN) quality standards. PLAN identified eighteen functions of a liaison team and provided details of quality standards within each function. These standards are either considered essential, expected or desirable. An accredited service is expected to meet 100% of essential standards, 80% of expected standards and 60% of desirable standards. Data were taken from a combination of sources including ABUHB policies, service managers and senior clinicians within both mental health and acute services.

**Result:**

When comparing the current liaison service provision in ABUHB, 30% of essential standards were not met and 21% were only somewhat met. Particular domains that were identifed as needing improvement included policies and procedures and urgent and emergency mental health care. 36% of expected standards were met with 41% not met. Notable domains that the service was performing poorly in included governance; induction, and providing teaching and support to acute colleagues. 89% of desirable standards were not met.

**Conclusion:**

The audit idenitifed that the current liaison service fails to meet core standards set out by RCPsych. This audit provides quantitative data to demonstrate that the liaison service is in need of improvement and investment. As a result, enhaving the current liaison service is now a priortity for the health board. A business case is being developed to consider enhancing the liaison service with a view to developing a Consultant led multidisciplinary team. The business case can use PLAN quality standards to make recommendations for improvements to the service.

